# Physical Activity, Sun Exposure, Vitamin D Intake and Perceived Stress in Italian Adults

**DOI:** 10.3390/nu15102301

**Published:** 2023-05-13

**Authors:** Bruno Trovato, Justyna Godos, Simone Varrasi, Federico Roggio, Sabrina Castellano, Giuseppe Musumeci

**Affiliations:** 1 Department of Biomedical and Biotechnological Sciences, Section of Anatomy, Histology and Movement Science, School of Medicine, University of Catania, Via S. Sofia 97, 95123 Catania, Italy; bruno.trovato@phd.unict.it (B.T.); federico.roggio@unict.it (F.R.); g.musumeci@unict.it (G.M.); 2 Department of Biomedical and Biotechnological Sciences, University of Catania, 95123 Catania, Italy; 3 Department of Educational Sciences, University of Catania, 95124 Catania, Italy; simonvarra@gmail.com (S.V.); sabrina.castellano@unict.it (S.C.); 4 Sport and Exercise Sciences Research Unit, Department of Psychology, Educational Science and Human Movement, University of Palermo, Via Giovanni Pascoli 6, 90144 Palermo, Italy

**Keywords:** physical activity, vitamin D, sun exposure, perceived stress, mental health, Mediterranean

## Abstract

The last decades of global development have, due to rapid urbanization, pressuring entire populations to changes in lifestyle and dietary habits, led to an increase in the prevalence of mental disorders, including stress. This study explored how lifestyle and dietary factors, such as physical activity, sun exposure, and vitamin D intake are related to perceived stress in a Mediterranean-based population. Physical activity level was evaluated using the international physical activity questionnaires (IPAQ), sun exposure was evaluated using the sunlight exposure measurement questionnaire (SEM-Q), and validated food frequency questionnaires (FFQs) were used to assess dietary intakes. The perceived stress of the study participants was evaluated using the perceived stress scale (PSS). Multivariate logistic regression models were used to test for potential associations. In the most adjusted model, an inverse association between physical activity level, sunlight exposure, vitamin D intake, and high perceived stress was found (OR = 0.72, 95% CI: 0.51, 1.00, OR = 0.72, 95% CI: 0.52, 0.99, OR = 0.69, 95% CI: 0.53, 0.89, respectively). However, when stratifying the population by level of physical activity, the retrieved associations with sunlight exposure and dietary vitamin D intake were significant only among those individuals reporting being moderately to highly physically active (OR = 0.16, 95% CI: 0.08, 0.33 and OR = 0.46, 95% CI: 0.28, 0.76, respectively), while results on low physically active participants were null. In conclusion, this study demonstrated that higher dietary intake of vitamin D and sunlight exposure are associated with a lower likelihood of having high perceived stress among physically active individuals.

## 1. Introduction

The first documented definition of stress in literature is the one given by Selye in 1956, defining stress as a psychological response of the body dealing with positive or negative non-specific demands [1]. Since then, researchers throughout the world failed to identify a rigorous definition of stress, providing misleading definitions of it [2]. The World Health Organization (WHO) defines stress as a state of worry or mental tension due to a difficult situation to challenge. Nowadays, stress is regarded as a series of experiences that generate frustration or anxiety due to a perceived threat to one’s security or the inability of an individual to cope with it [3]. Exposure to chronic or acute stressors endorses the release of catecholamines and cortisol that are fundamental for the fight-or-flight response [4]. However, when their levels remain high throughout the day, they can negatively affect psychophysiological well-being [4]. Stressful events are often more easily remembered, and there is a correlation between chronic and acute stress and morphological adaptation/alterations of the amygdala. This indicates that stress has the potential to actively modify a biological system [5]. Furthermore, chronic exposure to stress that elicits a maladaptive response can promote dysregulation of the immune system, inducing migraines and increasing the risk of cardiovascular pathologies, depression, and symptoms related to anxiety [6]. Therefore, targeting modifiable risk factors such as dietary habits and physical activity to improve the resilience of a person is fundamental [7].

Physical activity is one of the most useful strategies to manage stress without the risk of harming an individual with pharmacological treatment. The benefits of physical activity for both healthy and pathological people include reduced risk of metabolic [8], musculoskeletal [9], and neurological diseases [10]. Moreover, the positive effects of being physically active, even at levels below that recommended by the WHO, have also been demonstrated for mental health outcomes [11]. A possible beneficial mechanism through which physical activity may exert an action toward mental health (including tension, anger, and depressive feelings) is outdoor practice [12]. Such activity could increase exposure to sunlight, which can improve mood and has a positive association with cognitive function [13]. Moreover, skin exposure to sunlight starts the metabolism of vitamin D, due to the UVB ray that transforms the 7-dehydrocholesterol in pre-vitamin D3, isomerized later into vitamin D3 [14]. Several studies affirmed that deficiency in levels of vitamin D3 are associated with different health-threatening diseases, such as breast cancer [15], cardio-metabolic conditions [16], musculoskeletal pathologies [17,18], depression [19], and sleep disorders [20]. Low levels of vitamin D may also provoke deficits in strength and degeneration of glycolytic muscle fibers, thus reducing physical performance [21]. The only method to improve the levels of vitamin D, other than the exposition to sunlight, is with nutrition. Currently, in Europe, the intake of vitamin D through nutrition is low and its deficiency in the general population is a main concern for the public health system [22]. Considering that vitamin D supplementation can have positive effects in reducing symptoms of depression and anxiety [23], studying its levels in the general population and promoting a correct intake with nutrition is essential to try to manage stress from different points of view. The aim of this study is to understand the impact of physical activity, sunlight exposure, and vitamin D intake with nutrition on levels of perceived stress in a sample derived from the Italian general population.

## 2. Materials and Methods

### 2.1. Study Design and Population

The Mediterranean healthy eating, aging, and lifestyles (MEAL) study is an observational study aiming to assess the link between dietary habits, in the context of a cluster of lifestyle behaviors characterizing the Mediterranean basin, and non-communicable diseases. A complete protocol of the MEAL study has been published previously [24]. The cohort and baseline survey was established between 2014–2015 in southern Italy by randomly enrolling a sample of 2044 men and women aged 18 or more years old through the registered records of local general practitioners stratified by sex and 10-year age groups.

For the purpose of providing a specific relative precision of 5% (Type I error, 0.05; Type II error, 0.10), considering an anticipated 70% participation rate, the theoretical sample size was estimated to be 1500 individuals. In brief, a sample of 2405 individuals was invited to participate in the study, out of which 361 individuals declined, leaving 2044 participants as the final sample included, with a response rate of 85%. The aims of the study were exhaustively explained to all of the participants before the acceptance of participation; those who agreed provided a written informed consent. The study procedures were conducted in line with the Declaration of Helsinki (1989) of the World Medical Association. The study protocol has been evaluated and approved by the concerning ethical committee.

### 2.2. Physical Activity Assessment

The data were collected by trained personnel via face-to-face, computer-assisted interviews. The physical activity level was evaluated using the international physical activity questionnaires (IPAQ) [25]. The IPAQ consists of questions referring to five domains on time dedicated to physical activity in the last week. In brief, individuals are considered as having a “high physical activity level” if performing strenuous physical activity on no less than three days, reaching a minimum total physical activity of no less than 1500 MET minutes/w or ≥seven days of any combination of walking, moderate-intensity or vigorous-intensity activities reaching a minimum total physical activity of no less than 3000 MET minutes/w, while individuals performing ≥three days of vigorous-intensity activity of at least 20 min/d or ≥five days of moderate-intensity activity or walking of at least 30 min/d or ≥five days of any combination of walking, moderate-intensity or vigorous-intensity activities achieving a minimum of at least 600 MET minutes/w are considered as having “moderate physical activity level”. Finally, individuals who do not meet the criteria for being highly or moderately physically active are categorized as having a low physical activity level.

### 2.3. Sun Exposure Assessment

Information on sun exposure was evaluated using the sunlight exposure measurement questionnaire (SEM-Q) [26]. The tool consisted of 6 items investigating the occasions and level of exposure to sunlight through assigning different weights (ranging between 0 and 1) according to sun exposure (i.e., using sunscreen creams, % of body exposed, etc.). The final scoring algorithm was generated by multiplying the time (minutes) spent in the sun by the proportions of different domains and then divided into three groups, defined as low, medium, and high exposure.

### 2.4. Dietary Assessment

The dietary exposures were assessed through a long and a short version of food frequency questionnaires (FFQs), formerly tested for validity and reliability in Sicilian individuals [27,28]. The intake of seasonal foods was calculated based on the consumption during the time period of their availability and subsequently adjusted by their proportional intake over one year. Dietary intakes of macro- and micronutrients, as well as total energy intake, were determined using food composition tables of the Council for Research in Agriculture and Analysis of Agricultural Economy (CREA) [29]. In detail, the individual food consumption (in mL or g) was calculated for each individual by converting the standard portion sizes and different frequencies of consumption to 24 h intake. After, the databases were screened for average values of the energy content and macro- and micronutrients contained in each food (per 100 mL or g). Lastly, the energy and nutrient intake from foods was calculated by multiplying the content of each variable by the daily consumption of each food. FFQs with missing data or unreliable dietary intakes (<1000 or >6000 kcal/d) were not included in the analyses. Vitamin D data was calculated from dietary intakes (participants were asked to report eventual supplementation).

The quality of an individual’s diet was examined by calculating adherence to the Mediterranean diet using a validated literature-based score [30]. In brief, two points were assigned to the highest category of intake of foods typical of the Mediterranean diet (such as vegetables, fruits, legumes, cereals, fish), one point for the middle category, and 0 points for the lowest category of intake. On the contrary, two points were assigned for the lowest category of intake of foods not characteristic of the Mediterranean diet (such as meat and dairy products), one point for the middle category, and 0 points for the highest category of intake. Moderate alcohol intake and regular use of olive oil contributed to the higher adherence. The final score consists of nine food groups with a minimum of 0 points (lowest adherence) and maximum of 18 points (highest adherence), and individuals catalogued in the subsequent tertiles: (i) low, (ii) medium, and (iii) high adherence to the Mediterranean diet [31].

### 2.5. Covariate Assessment

In addition to dietary data, information on factors potentially associated with the investigated exposures and outcomes was collected. Data on sex, age, marital and educational (the highest educational degree achieved) status, and smoking status were collected. Marital status was catalogued as (i) unmarried/widowed or (ii) married. Educational status was catalogued as (i) low (primary/secondary), (ii) medium (high school), and (iii) high (university). Smoking status was catalogued as (i) non-smoker, (ii) ex-smoker, and (iii) current smoker. Eating habits comprised questions on skipping breakfast, daily snacking, skipping dinner, and out-of-home eating, with answers categorized as (i) always/often and (ii) seldom/never. Finally, body mass index (BMI) was categorized as normal weight (BMI < 25 kg/m^2^), overweight (BMI 25 to 29.9 kg/m^2^), and obese (BMI ≥ 30 kg/m^2^) [32].

### 2.6. Perceived Stress Assessment

The stress symptoms of the study participants were assessed using the perceived stress scale (PSS) [33]. Briefly, the PSS is a 14-item questionnaire used to evaluate perceived stress by examining the level to which situations in an individual’s life are perceived as stressful. Each query has five possible answers ranging from zero to four (0 = never, 1 = almost never, 2 = sometimes, 3 = often, and 4 = always). The final score is the sum of the scores of 14 queries and it ranges from 0 (minimum) to 56 (maximum). The sex-specific median value was considered as a cut-off point to define high or low perceived stress.

### 2.7. Statistical Analysis

After excluding missing entries from tools or data collection, a total of 1728 individuals were included in the analysis. Categorical variables are reported as frequencies of occurrence and percentages, with the Chi-squared test used to assess differences between physical activity levels. Continuous variables are reported as mean and standard deviations (SDs), with the ANOVA test used to test differences between groups. Multivariate logistic regression models were calculated to determine the cross-sectional association between physical activity level, dietary vitamin D intake, sunlight exposure, and perceived stress. Odds ratios (ORs) and 95% confidence intervals (CIs) were estimated for different models adjusted for potential confounders, including background characteristics and Mediterranean diet adherence as a proxy of diet quality. An additional analysis via stratifying the sample by low and medium/high category for each variable of exposure was further investigated to test their influencing role on each other. The reported *p* values were based on two-sided tests and compared to a significance level of 5%. All the statistical calculations were performed using SPSS 21 (SPSS Inc., Chicago, IL, USA) software.

## 3. Results

The background characteristics of the study sample by level of physical activity are presented in Table 1. Among individuals reporting higher levels of physical activity, there was a higher proportion of younger men, with a higher educational level and more current smokers than those reporting lower physical activity. Concerning eating habits, among those more physically active, there also was a higher prevalence of individuals reporting daily snacking and out-of-home eating, as well as a higher adherence to the Mediterranean diet.

**Figure 1:**
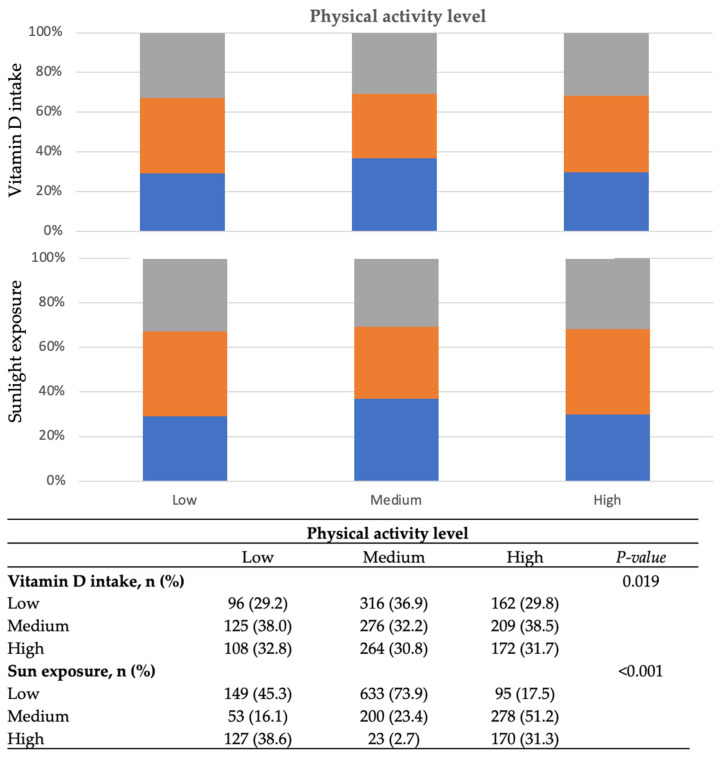
Distribution of dietary vitamin D intake, sunlight exposure, and physical activity level in the study sample (*n* = 1728). Gray color identifies low category, orange color identifies medium, and blue color identifies high category of vitamin D intake and sunlight exposure.

The distribution of dietary vitamin D intake and sunlight exposure by the level of physical activity is presented in Figure 1. There was a significant difference in the distribution of both variables; interestingly, there was a higher proportion of individuals reporting low vitamin D intake and sunlight exposure in the medium category of physical activity level, while higher vitamin D intake and sunlight exposure was more evident in both low and high physical activity level groups, suggesting no clear trends in the distribution of these variables.

Table 2 presents the results of the logistic regression analyses assessing the association between the variables of interest and perceived stress. After adjusting for potential confounding factors (including diet quality assessed as higher adherence to the Mediterranean diet), individuals in the highest group of physical activity level (OR = 0.72, 95% CI: 0.51, 1.00), sunlight exposure (OR = 0.72, 95% CI: 0.52, 0.99), and vitamin D intake (OR = 0.69, 95% CI: 0.53, 0.89) were less likely to report perceived stress compared to those in the lowest groups; similar findings were retrieved for the medium category of physical activity level and sunlight exposure (Table 2).

**Figure 2:**
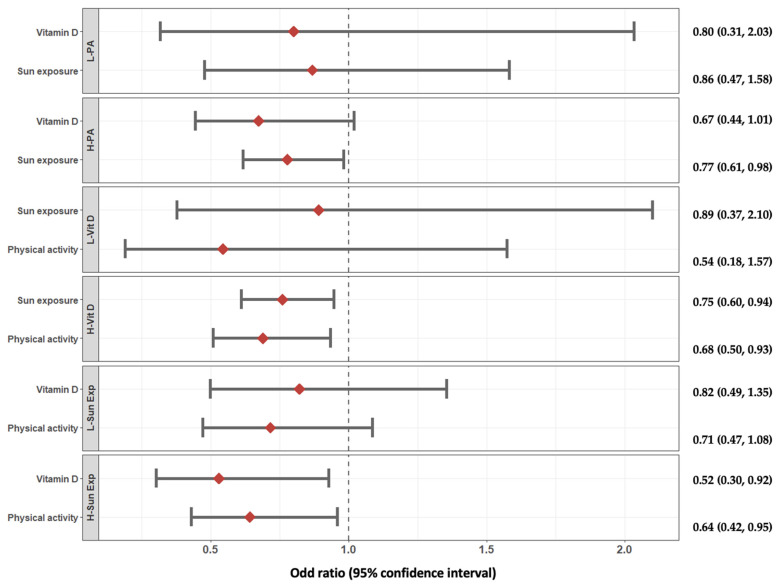
Odds ratio (ORs) and 95% confidence intervals (CIs) of the association between difference level of exposure to physical activity, vitamin D intake, and sunlight exposure and perceived stress. Multivariate model was adjusted for BMI (normal, overweight, obese), sex, educational status (low, medium, high), smoking status (never, current, former), age (continuous, years), eating habits (skipping breakfast, daily snacking, skipping dinner, out of home eating), marital status (unmarried/widowed, married), and adherence to the Mediterranean diet. L-PA, low physical activity; H-PA, high physical activity; L-Vit D, low vitamin D intake; H-Vit D, high vitamin D intake; L-Sun Exp, low sunlight exposure; H-Sun Exp, high sunlight exposure.

The stratification by low and medium/high category of each variable of exposure to test their influencing role on each other is presented in Figure 2. Interestingly, neither vitamin D intake nor sunlight exposure was significantly associated with perceived stress in the low physical activity group, while individuals reporting medium-to-high physical activity levels and being in the highest category of exposure for both vitamin D intake and sunlight exposure were associated with lower odds of having perceived stress (OR = 0.67, 95% CI: 0.44, 1.01 and OR = 0.77, 95% CI: 0.61, 0.98, respectively; Figure 2). Similarly, when considering low exposure to dietary vitamin D and sunlight exposure, there were no evidence associations for the other variables, while in individuals with higher vitamin D intake there was a significant association of both high physical activity and high sunlight exposure (OR = 0.68, 95% CI: 0.50, 0.93 and OR = 0.75, 95% CI: 0.60, 0.94, respectively); as additionally, individuals with higher sunlight exposure had a significant association of both high physical activity and high vitamin D intake with lower likelihood of having perceived stress (OR = 0.64, 95% CI: 0.42, 0.95 and OR = 0.52, 95% CI: 0.30, 0.92, respectively; Figure 2).

## 4. Discussion

In this study, the association between physical activity level, sunlight exposure, dietary vitamin D intake, and perceived stress levels was investigated in a sample of Italian adults. An inverse association between all the variables of exposure and perceived stress was found. However, when stratifying the population by level of physical activity, vitamin D intake, and sunlight exposure, the retrieved associations of each variable with perceived stress were significant only among individuals with higher exposure to the others, suggesting a strict relation between them.

Physical activity has been positively associated with the reduction of musculoskeletal [34] and major non-communicable diseases [35,36]; furthermore, mental conditions [37] can benefit from its regular and tailored practice. Previous studies reported substantial benefits of physical activity on psychological conditions, such as burnout [38,39], anxiety [40,41], or depression [42]. Concerning perceived stress, a previous study conducted on a sample of 537 college students showed that vigorous and moderate physical activity were inversely associated with perceived stress [43]. In line with our results, similar findings have also been reported in intervention studies. A meta-analysis of 13 randomized controlled trials showed that exercise in evidence-based treatments for individuals with stress or anxiety disorders was able to improve symptoms [44].

The stress reduction related to an increase in physical activity detected in our sample could be explained through several mechanisms. Physical activity promotes the secretion of β-Endorphin, a hormone that can positively influence mood states [45]. Moreover, some physical activity can be performed outdoors, thus increasing exposure to sunlight. There is consistent evidence from the scientific literature that performing physical activity outdoors has a higher positive impact on mood and mental well-being compared to indoor activities, possibly related to exposure to sunlight [12,46]. Additionally, in the present study, we found that sun exposure was associated with lower perceived stress, and that the association was significant among individuals with moderate-to-high physical activity levels. In line with our findings, in a study conducted on 948 Korean adults, people with less exposure to sunlight had significantly higher levels of perceived stress than those who were more exposed to sunlight [47]. Mechanistically, it has been hypothesized that mood regulation by the light needs a pathway independent of the suprachiasmatic nucleus that links intrinsically photosensitive retinal ganglion cells to the perihabenular nucleus, which is a mood regulator [48]. Furthermore, sunlight is fundamental for vitamin D metabolism, with UVB rays transforming 7-dehydrocholesterol to pre-vitamin D3, then isomerizing into vitamin D [14]: the positive effects of sunlight on stress could be explained by enhancement in vitamin D levels. We further found that also higher intake of vitamin D was inversely associated with perceived stress, especially in individuals reporting moderate-to-high physical activity levels. A previous investigation conducted on Northern American adults showed that those having lower serum 25(OH)D concentrations had poorer mental health and psychosocial stress compared to those with higher concentrations of serum 25(OH)D [49]. In fact, vitamin D receptors (VDR) and their activating enzymes are present in various regions of the human brain and 25(OH)D can reach the central nervous system by crossing the blood–brain barrier, indicating that it could potentially have autocrine-paracrine functions within the human brain [50]. VDR and 1a-hydroxylase have been found to be distributed in the human brain, both in glial cells and neurons, in a layer-specific pattern, especially in the hypothalamus and neurons within the substantia nigra [50]. Stress activates the hypothalamic–pituitary–adrenal axis (HPA) [51], which functions in connection with the autonomic nervous system and the immune system in managing the hormonal and inflammatory responses to stress [52]. The HPA activation promotes the release of corticotropin-releasing hormone in the hypothalamus and the secretion of adrenocorticotropin from the pituitary cells, mediating adaptive responses that are necessary for survival [52]. However, chronic stress may elicit harmful changes that can bring an alteration of the HPA fostering long-term pathological alterations [53]. It was shown that one of the metabolites of vitamin D,1,25-dihydroxyvitamin D3 has neuroprotective effects, upregulating neurotrophin-3 and neurotrophin-4 [54], and nerve growth factors [55] that can be found in the hippocampus and neocortex. Furthermore, vitamin D influences inflammatory pathways that have been associated with depression [56] by activating anti-inflammatory pathways through VDR-mediated gene transcription and downregulating autoimmune mechanisms that produce proinflammatory cytokines [54,57]. These underlying physiological mechanisms could explain our findings of an inverse association between lower levels of perceived stress in people with higher vitamin D intake.

To the best of our knowledge, this is the first study that analyzes the association between physical activity, sun exposure, vitamin D, and perceived stress in a sample of the general Italian population. Nonetheless, despite the novelty of this study, the results may be subjected to some limitations. Considering the cross-sectional nature of the analysis, the reverse causation cannot be ruled out. Another limitation concerns the dietary assessment method, as the use of FFQs may potentially under- or overestimate food intake due to recall bias. Additionally, despite adjusting for multiple confounding factors, the results may have been influenced by unmeasured variables. Another limitation may be related to the lack of information on whether individuals are primarily engaged in indoor or outdoor sports and, thus, directly exposed to more or less sunlight. The PSS is a good screening tool to assess the prevalence of stress symptoms in large sample sizes; however, it may not be deemed as a clinical evaluation, and thus may over- or underestimate the outcome prevalence. Finally, the findings of this study may not be generalizable to other populations as several factors may affect the intensity of sun exposure, including but not limited to solar zenith angle, altitude, or skin type.

## 5. Conclusions

In conclusion, the results of this study highlight that higher sun exposure and dietary intake of vitamin D are associated with a lower likelihood of having high perceived stress among physically active individuals.

## Figures and Tables

**Table 1:** Demographic characteristics of the study participants according to physical activity level (*n* = 1728).

		Physical Activity Level		
	Low (*n* = 329)	Medium (*n* = 856)	High (*n* = 543)	*p*-Value
**Age, mean (SD)**	55 (19.23)	47 (17.24)	44 (14.77)	<0.001
**Age groups, *n* (%)**				<0.001
18–34	36 (20.0)	51 (22.9)	132 (29.5)	
35–49	36 (20.0)	54 (24.2)	174 (38.8)	
50–64	40 (22.2)	77 (34.5)	105 (23.4)	
65+	68 (37.8)	41 (18.4)	37 (8.3)	
**Sex, *n* (%)**				<0.001
Men	102 (31.0)	338 (39.5)	285 (52.5)	
Women	227 (69.0)	518 (60.5)	258 (47.5)	
**Smoking status, *n* (%)**				0.002
Never	213 (64.7)	527 (61.6)	340 (62.6)	
Current	76 (23.1)	195 (22.8)	155 (28.5)	
Former	40 (12.2)	134 (15.7)	48 (8.8)	
**BMI categories, *n* (%)**				<0.001
Normal weight	101 (35.1)	390 (49.6)	269 (51.8)	
Overweight	101 (35.1)	270 (34.3)	192 (37.0)	
Obese	86 (29.9)	127 (16.1)	58 (11.2)	
**Educational level, *n* (%)**				<0.001
Low	185 (56.2)	228 (26.6)	153 (28.2)	
Medium	79 (24.0)	327 (38.2)	264 (48.6)	
High	65 (19.8)	301 (35.2)	126 (23.2)	
**Marital status, *n* (%)**				0.177
Unmarried/widowed	127 (38.6)	362 (42.3)	204 (37.6)	
Married	202 (61.4)	494 (57.7)	339 (62.4)	
**Eating habits, *n* (%)**				
Skipping breakfast	71 (21.6)	197 (23.0)	148 (27.3)	0.098
Daily snacking	230 (69.9)	693 (81.0)	401 (73.8)	<0.001
Skipping dinner	13 (4.0)	64 (7.5)	36 (6.6)	0.089
Out of home eating	97 (29.5)	479 (56.0)	340 (62.6)	<0.001
**Mediterranean diet adherence, *n* (%)**				0.009
Low	185 (56.2)	494 (57.7)	264 (48.6)	
Medium	117 (35.6)	288 (33.6)	212 (39.0)	
High	27 (8.2)	74 (8.6)	67 (12.3)	

**Table 2:** Odds ratios (ORs) and 95% confidence intervals (CIs) of the association between physical activity level, sunlight exposure, vitamin D intake, and perceived stress.

	**Perceived Stress, OR (95% CI)**		
	**Low**	**Medium**	**High**
**Physical activity level**			
Unadjusted	1	0.68 (0.51, 0.91)	0.80 (0.60, 1.08)
Model 1 *	1	0.65 (0.47, 0.89)	0.73 (0.53, 1.02)
Model 2 **	1	0.64 (0.46, 0.88)	0.72 (0.51, 1.00)
**Vitamin D intake**			
Unadjusted	1	0.90 (0.71, 1.14)	0.81 (0.64, 1.02)
Model 1 *	1	0.82 (0.64, 1.05)	0.72 (0.56, 0.94)
Model 2 **	1	0.80 (0.63, 1.03)	0.69 (0.53, 0.89)
**Sun exposure**			
Unadjusted	1	0.81 (0.63, 1.02)	0.74 (0.55, 1.00)
Model 1 *	1	0.76 (0.59, 0.99)	0.73 (0.53, 1.01)
Model 2 **	1	0.76 (0.59, 0.98)	0.72 (0.52, 0.99)

* Multivariate model 1 was adjusted for BMI (normal, overweight, obese), sex, educational status (low, medium, high), smoking status (never, current, former), age (continuous, years), eating habits (skipping breakfast, daily snacking, skipping dinner, out of home eating), marital status (unmarried/widowed, married). ** Multivariate model 2 was further adjusted for adherence to the Mediterranean diet.

## Data Availability

The data that support the findings of this study are available upon reasonable request.
